# Breaking down walls: a qualitative evaluation of perceived emergency department delays for patients transferred with ST-elevation myocardial infarction

**DOI:** 10.1186/s12873-020-00355-6

**Published:** 2020-08-06

**Authors:** Michael J. Ward, Timothy J. Vogus, Kemberlee Bonnet, Kelly Moser, David Schlundt, Sunil Kripalani

**Affiliations:** 1 Department of Emergency Medicine, Vanderbilt University Medical Center, VA Tennessee Valley Healthcare System, 1313 21st Ave. Oxford House #330, Nashville, TN 37203 USA; 2 Center for Clinical Quality and Implementation Research, Vanderbilt University Medical Center, Nashville, USA; 3 Vanderbilt University Owen Graduate School of Management, Nashville, USA; 4 Department of Psychology, Vanderbilt University, Nashville, USA; 5 Department of Emergency Medicine, Vanderbilt University Medical Center, Nashville, USA; 6 Department of Internal Medicine, Vanderbilt University Medical Center, Nashville, USA

**Keywords:** ST-elevation myocardial infarction, Inter-facility transfer, Emergency medicine, Care coordination, Qualitative methods

## Abstract

**Background:**

Despite regionalization efforts, delays at transferring hospitals for patients transferred with ST-elevation myocardial infarction (STEMI) for primary percutaneous coronary intervention (PCI) persist. These delays primarily occur in the emergency department (ED), and are associated with increased mortality. We sought to use qualitative methods to understand staff and clinician perceptions underlying these delays.

**Methods:**

We conducted semi-structured interviews at 3 EDs that routinely transfer STEMI patients to identify staff perceptions of delays and potential interventions. Interviews were recorded, transcribed, coded, and analyzed using an iterative inductive-deductive approach to build and refine a list of themes and subthemes, and identify supporting quotes.

**Results:**

We interviewed 43 ED staff (staff, nurses, and physicians) and identified 3 major themes influencing inter-facility transfers of STEMI patients: 1) Processes, 2) Communication; and 3) Resources. Standardized processes (i.e., protocols) reduce uncertainty and can mobilize resources. Use of performance benchmarks can motivate staff but are frequently focused on internal, not inter-organizational performance. Direct use ofcommunication between ORGANIZATIONS can process uncertainty and expedite care. Record sharing and regular post-transfer communication could provide opportunities to discuss and learn from delays and increase professional satisfaction. Finally, characteristics of resources that enhanced their capacity, clarity, experience, and reliability were identified as contributing to timely transfers.

**Conclusions:**

Processes, communication, and resources were identified as modifying inter-facility transfer timeliness. Potential quality improvement strategies include ongoing updates of protocols within and between organizations to account for changes, enhanced post-transfer feedback between organizations, shared medical records, and designated roles for coordination.

## Background

Despite decreasing emergency department (ED) visits for ST-elevation myocardial infarction (STEMI) [1], nearly 1 in 5 ED visits with STEMI result in transfer to another facility and both the absolute number and rate of inter-facility transfer are increasing [2]. Timely access to the preferred treatment, primary percutaneous coronary intervention (PCI) is associated with improved outcomes [3], particularly for transfers [4]. While the American College of Cardiology Foundation/American Heart Association (AHA) has a recommendation for inter-facility transfer from a non-PCI to PCI facility with a first medical contact-to-device of ≤120 min (Class I, Level of Evidence B), these are difficult to achieve for transfers even under conditions such as those that occurred during the AHA’s Mission:Lifeline quality improvement program and subsequent Accelerator program [5–7]. Transferring EDs are recognized as the primary source of delay [4], and prior retrospective analyses have identified the time spent at transferring EDs [8–10], rurality, and PCI hospital volume as contributing to delays [5]. However, understanding of the perceived mechanisms of these underlying delays remains limited.

Qualitative research richly captures the process dynamics surrounding more and less successful transfers. Given the common delays associated with inter-facility transfer for patients with STEMI, exploring why these delays persist through the use of qualitative methods may facilitate an identification and description of the mechanisms underlying observed delays.

We sought to examine the perceptions of ED staff and clinical providers who have variable access to PCI capabilities and must subsequently perform inter-facility transfers in order to access such care. We sought to identify perceived facilitators and barriers to the provision of timely care at the transferring ED to gain an in-depth understanding of the transfer process as well as to provide a foundation for more refined quantitative empirical research in the future.

## Methods

This research was conducted in compliance with the COREQ (COnsolidated criteria for REporting Qualitative research) checklist [11]. This study was approved by the Vanderbilt University Medical Center (VUMC) institutional review board (IRB) and participants provided written informed consent. Participating sites were exempt from local IRB approval as these sites were determined not to be engaged in research.

### Research team

Two investigators, one male (MJW) and one female (KM) conducted the interviews. Investigator 1 is a practicing emergency physician and mixed methods researcher with an MD, PhD. Investigator 2 is a trained research coordinator in emergency medicine. Both were trained by senior qualitative researchers to conduct interviews. Training included mentored development of the interview guide, pilot testing of the interview guide with three volunteers.

### Selection of participants

We conducted and analyzed semi-structured interviews of staff (administrative, nursing, and emergency physicians) at three EDs in the metropolitan Nashville, TN area that transferred STEMI patients to surrounding hospitals for primary PCI. Characteristics of these EDs can be seen in Table 1. All three sites have annual ED volumes less than 40,000 visits per year with varying access to primary PCI. At Facility A, the cardiac catherization laboratory was not open during evening and weekend hours and frequently did not have consistent primary PCI availability during daytime hours. At Facility B, 24/7 cardiology services were available, but transfers continued to occur for lack of capacity or patient complexity reasons. At Facility C, primary PCI capabilities were available during business hours but not after hours.

**Table 1:** Characteristics of the facilities included in the study

Site	Type of Facility	Rural/Urban	Approximate Annual ED Volume	Staff Interviewed
Facility A	Community	Urban	33,000	• ED Nurses (7) • Emergency Physicians (4)
Facility B	Community	Suburban	30,000	• ED Nurses (7) • Emergency Physicians (3) • Staff (3)
Facility C	Federal	Urban	25,000	• ED Nurses (12) • Emergency Physicians (4) • Staff (3)

Initial interview participants were recruited through clinical leadership (e.g., nurse managers and medical directors) at each facility and then subsequent participants were identified through snowball sampling. Interviewees were included if they were actively employed in the ED at the time of interview regardless of seniority or experience level in order to provide a variety of perspectives. In addition, when working clinically in the ED, these individuals could be asked to participate in a STEMI transfer at any time so their knowledge at the time of interview reflected the current state of the ED. No relationship was established prior to interview and interview subjects were met at the time of interview. Beyond the rationale for the study, personal investigator perspectives about STEMI transfer were intentionally left out of the interview so as not to bias interviewee responses. Participants were interviewed over a three-month period and were offered the opportunity to participate in a drawing for a $100 gift card. In addition, letters were written to administrative supervisors recognizing individual staff efforts to participate in research.

### Procedure

The goal of the interview, which was shared with interviewees, was to elicit discussion of perceived facilitators and barriers to rapid and safe inter-facility transfers for STEMI particularly when there was variability in the conduct and use of transfer. We decided to use semi-structured individual interviews rather than focus groups for two reasons. First, the content of interviews and responses could be perceived as potentially sensitive so we decided to use a semi-structured individual interview approach for qualitative evaluation rather than focus groups in order to foster psychological safety through the use of smaller groups [12]. Second, individual interviews are logistically more feasible compared with coordinating multiple individuals. We developed questions about the STEMI transfer process to understand how transfers were conducted, perceived facilitators and barriers to timely and safe transfer, and organizational influence on the transfers. Questions were open-ended, but interviewers engaged in follow-up questions to encourage clarification, elaboration, when encountering a new idea. We developed and piloted the written semi-structured interview guide with three individuals to ensure fidelity of the interviewers, revise questions, and to ensure a maximum length of 30 min. The final interview guide can be seen in Supplemental Figure 1. All interviews were conducted in English, audio recorded, and transcribed for analysis. Field notes were recorded during interviews. We targeted an estimated 10–15 interviews per site in order to reach theoretical saturation, the point at which additional data do not identify new themes [13]. Transcripts were imported into Excel spreadsheets, which were used to manage and organize the coding. Following completion of the coding, we examined the coded transcripts using SPSS v25 (IBM, Inc. Armonk, NY) to calculate frequencies of codes.

### Analysis

Two investigators (KB and MJW) developed a preliminary coding framework for the transcripts including broader themes of transfers along with specific codes identified during interviews. Codes were then developed about staffing roles and experience, processes, facilitators and barriers, and recommended interventions. Coding was conducted in an iterative inductive-deductive approach [14]. Deductively, we created general categories to reflect the flow of events, the people involved, and the problems encountered during STEMI transfers. Specific subcategories were created inductively from the interview transcripts and were organized hierarchically. We piloted this system using a subset of seven of the transcripts and subsequently revised the coding system until all coders felt that no new codes were needed. As patient clinical outcomes are not typically directly available to ED providers, we focused on the use of feedback delivered to clinical providers as a surrogate for outcomes. New codes were added if applicable to more than one interviewee and could not be identified elsewhere. Additional transcripts were analyzed until agreement between the investigators occurred that no additional new themes and codes were identified (i.e., saturation). These transcripts were then re-reviewed with the final coding framework which was then applied to the remaining transcripts. From prior qualitative experience with ED-based transfers [15], we allowed up to seven codes to be applied per quote. Two investigators reviewed every transcript independently and met to agree upon final coding of each quote. The final coding framework was organized as follows: 1) participant details; 2) STEMI experience; 3) Process of recognizing a STEMI; 4) Roles; 5) Time of day; 6) Transfer Process; 7) Facilitators and barriers in transfer; 8) Perception of experience; 9) Comparison between facility goals and philosophies; and 10) Interventions.

To facilitate analysis, quotes were sorted by code. Calculated code frequencies were used to revise the final organization of the themes and subthemes structure, process, outcome framework to finalize the themes and subthemes. Quotes corresponding to codes were sorted by category and organized into themes and subthemes to understand and identify representative quotes demonstrating facilitators and barriers of the inter-facility transfer process. This organization was iteratively designed, proposed to the investigator team and revised until consensus was achieved. Representative quotes were then identified from codes occurring at higher frequencies.

Participant checking was conducted indirectly in interviews with staff to verify or validate perceptions and was conducted directly with ED leadership at the conclusion of interviews at each site. ED leadership was provided an opportunity to give feedback about findings at this time. Given the variable nature of shift work in the ED, transcripts were not returned to interviewees for comment. However, investigators met with ED leadership to review findings, while protecting the identities of the interviewees, and to comment on the validity of identified themes and subthemes.

## Results

**Fig. 1:**
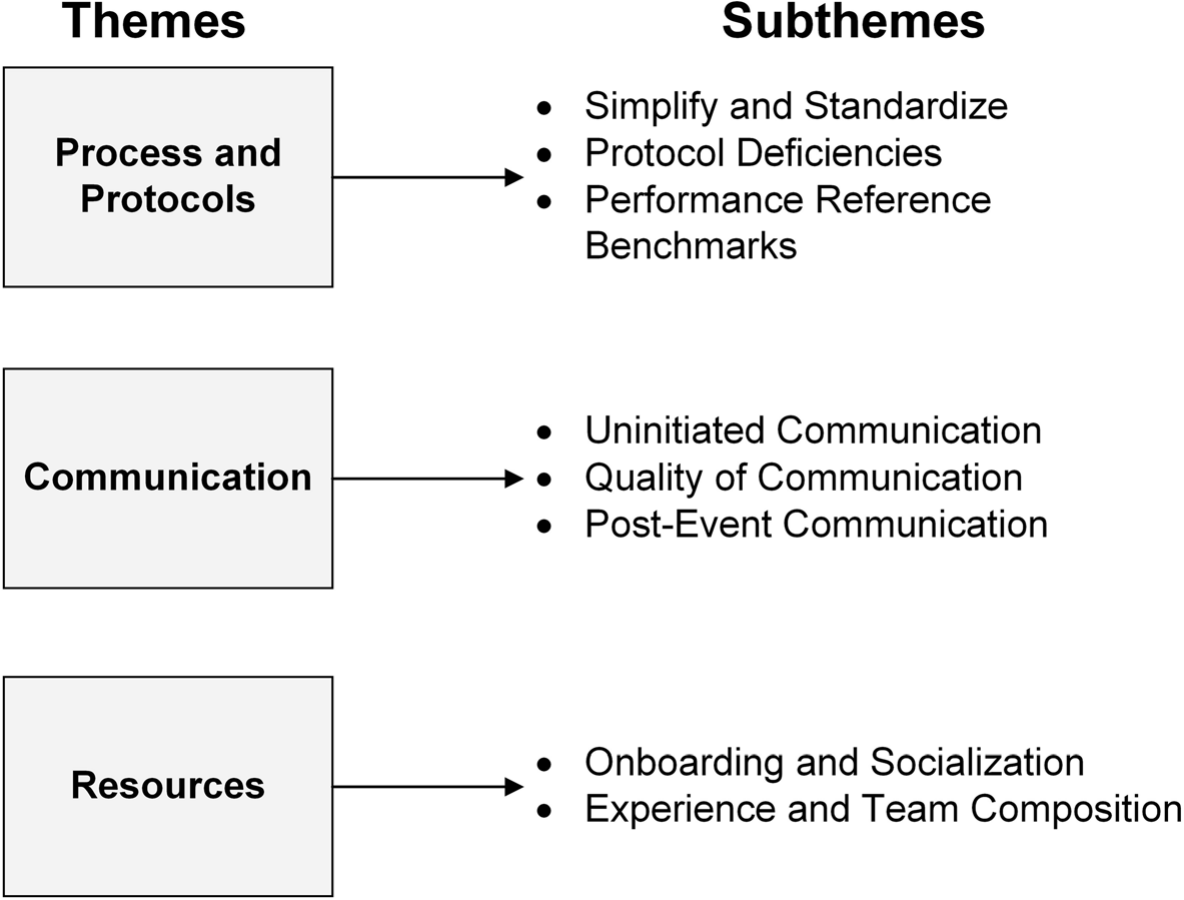
Finalized organization of themes and subthemes

We approached 43 individuals, all of whom agreed to be interviewed. All interviews were conducted in the respective EDs in which interviewees worked and non-participants were not allowed to attend so as to allow participants to remain candid. As many ED work spaces are open to staff, interviews were paused if non-participants entered. No repeat interviews were conducted. Among interviewees, there were 26 ED nurses, 11 emergency physicians, and six medical technologists or administrative support staff. Our revised organization of themes and subthemes are organized visually in Fig. 1. Themes included Process and Protocols, Communication, and Resources. Below, we discuss the finalized themes and subthemes using representative quotes.

The first theme of Process and Protocols included the subthemes of Simplify and Standardize, Protocol Deficiencies, and Performance Reference Benchmarks. Inter-facility and inter-agency agreements were recognized as a foundational component of transferring STEMI patients that facilitate the transfer process.

*[The PCI Center] ask the basic information … It's super straight forward. You just call a number and give information. – Nurse, Facility A*

*Because we're computerized sometimes the orders aren't in so there's a lot of verbal orders. That's why we made a very standardized protocol check sheet, so we pull that out that way it's right there. – Emergency Physician, Facility A*

*The communication was simple. You knew who to call. – Emergency Physician, Facility C*

*Quickly activating the STEMI protocol just to get all your resources moving and in place. That will trigger a phone call conversation with the transfer center and with the accepting physician. – Emergency Physician, Facility C*

Despite availability at all three sites, practical barriers to regular use and adherence to these protocols during the process of transfers were found due to incompleteness, ambiguity, and accessibility. Thus, we identified the subtheme of Protocol Deficiencies.

*We have different facilities get us those physicians on the line in different manners. There is a difference between which facility you call and how long you can expect to have somebody to speak with. – Emergency Physician, Facility B*

*What usually does change is after that once they decide the STEMI’s actually happening, what do we do now? Which always seems to be different depending on the situation. Charges [Nurses] aren't sure if they … Oh do they go here? Do we send them to [PCI Center]? Do we need to pick up the [cath lab] phone? How do we call cardiology? Kind of depending on who the charge nurse is or, which attending's on. That situation is always kind of is up in the air. – ED Technician, Facility C*

*I'm sure that there is [Referring to a protocol] somewhere but I don't whip it out on a day to day basis and go, "Oh my goodness, what do I do next?" Because you hope that your ER staff is a little bit proactive and a little bit ahead of needing to pull out a piece of paper to figure out what to do next. – Nurse, Facility C*

The final subtheme under Process and Protocols was Performance Benchmarks. This subtheme refers to the use of performance goals that are either set internally by the organization or externally (e.g., Joint Commission). The use of performance goals to enhance staff awareness of desired targets was recognized as a potential motivating source of internal competition and positive staff recognition.

*I think it's just been something that's been harped on, even outside the hospital just as a specialty and as a medical community it's been prioritized highly. I think the benchmarks that are given with door to needle time, door to balloon time, things like this, I think that metric does push the hospital which in turn pushes protocols and making things this is the way it should be done – Emergency Physician, Facility B*

*We want to beat the clock. Let's do this in 29 minutes. Forget 90 minutes we want to get it done fast. – Nurse, Facility C*

*We're always under our timeframe and one thing. Normally people get a ‘59 minute,’ which is like a little courtesy and a pat on the back. It makes them really want to move fast and get this done. Sometimes we don't get those. That's sad. It's a credit for an hour of time that you can use at your leisure like to come in late or take an hour early on a day that it's not, so you're given a little encouragement to beat the timeframe and get a patient to where he's supposed to be. – Nurse, Facility C*

Despite the recognition of performance benchmarks as potentially positive influence on staff performance, we also found that the focus on metrics was not always shared between organizations that transfer and receive STEMI patients.

*I know PCI staff is always very interested in time, and time to balloon, and ultimately, their metrics, which is not to say that we're not concerned with trying to get those patients in that situation the appropriate person or balloon in time, but that's certainly something that comes through when you talk to PCI or cath lab nurses – Emergency Physician, Facility C*

*I don't know [PCI Center] goals, so I don't know that there's any difference, I don't know. I'm sure there's a standard that we have to answer to, like the joint commission or something, because they have an EKG within five or ten minutes, or they would get them to the cath lab, because it's like, door-to-balloon. – Nurse, Facility C*

The next theme was Communication with three associated subthemes: Uninitiated, Quality, and Lack of Post-Event communication. We found that each subtheme was applicable to each stage of the transfer process, (before, during, or after). Uninitiated communication can occur when a staff member or clinician who has something to say or should communicate with others but does not do so. We found that this deficiency in communication occurred prior to the transfer taking place.

*A doctor may say, "This person needs to go," but doesn't let the nurse know. – Nurse, Facility A*

*I think the biggest thing is probably communication about who should alert the nurse that the cath lab's ready. – Nurse, Facility C*

High quality communication is when communication occurs both prior to and during the transfer and is timely, sufficiently detailed, mutual, and contributes to rapid treatment.

*We're going to call the cath lab, the cath lab charge [nurse] will call you back when they're ready and they'll call you back on their phone. It would be a little more proactive while the communications that so we all say, "It looks like it's going to be 9:30 before the cath lab is in" and it's 8:45. So we have a general idea versus just kind of appear, "Okay, send the patient" because they're driving in and it'd be nice to have a little bit more of a dynamic communication. – Emergency Physician, Facility C*

*There's a lot of moving parts. Getting the medications, having them quickly available, having an experienced team that is anticipating what you're going to do is very helpful. I think probably the two biggest factors are going in terms of be being able to have the communication with the accepting physician in a timely manner and having a transport team or available transport quickly. – Emergency Physician, Facility C*

Alternatively, low quality communication is disconnected from action and may get lost amidst a stream of tasks, and, consequently, require repeating information, and may delay timely care.

*Oftentimes we end up giving report twice….Sometimes I give report to the transfer center and it stops there. Other times I give a brief report to the transfer center and then we have to call either the ER or the unit to give report essentially a second time. – Nurse, Facility B*

*There was one STEMI that was right around shift change. Which was … the decision was made to take him over to [PCI Center], but there was a miscommunication on whether they wanted cardiology to come over here and see him or if we were supposed to take him straight over to [PCI Center] and with the nurses changing shift it kind of, almost, fell through a little bit. Obviously they didn't forget that there was a STEMI going on but I think the communication got slowed down and it might have delayed the care for that patient. – ED Technician, Facility C*

*We'll start to try to talk, they talk over us, which granted, they have their own thing they have to do. I get that, but you don't have to be rude… they don't even want to hear what we have to say, half the time. – Nurse, Facility C*

Post-Event communication was the last subtheme and referred to communication after the transfer predominately involving patient outcomes. Staff and clinicians were much more likely to speak about the lack of Post-Event communication rather than its presence following transfers. Staff recognized that Post-Event communication affected the transfer process and subsequent performance rather than changing an individual transfer.

*I think it would be good to know what they found, and what was done, just from a learning perspective. Because that way, the nurses that are involved will know, "That's what this looks like." – Nurse, Facility A*

*I don't know what happens with my patients once I transfer them out. I'd like to know what was done, what was found. I never get that information. I'd like to have that. – Emergency Physician, Facility A*

*That would be cool, just to give us feedback. Like "hey, on your guy's end this took a little longer than we like it to, maybe next time". Yeah, that would be good. I feel like a lot of times we don't know. I just fax stuff off and I have no clue if it actually gets there to be honest. I get my confirm fax, but I'm like, how do I know that the right person got it on the other side. – ED Technician, Facility B*

*I don't really get any feedback from a process standpoint on transfers. All that would be internal here on us recognizing that, "Hey, we could've done better on this." I don't think that process is looked at very closely from a transfer standpoint. – ED Technician, Facility B*

The last theme we identified was Resources. Resources can be human (e.g., staff, nurses, and physicians) or facility resources (e.g., EKG machine, or catheterization laboratory). When discussing human resources, issues involving the development and integration of team members within the broader team and organization were salient. Alternatively, when discussing facility resources, characteristics about their use were identified as relevant to transfers. The first subtheme was Onboarding and Socialization. This involves the process of bringing on new staff into the larger organization (i.e., hospital), incorporation into the ED, and their training in the process of transfers, particularly protocols:

*If we had new people, not new providers, but new nursing staff or people that weren't familiar with our processes. Some people work at a place that just has a continuous 24/7 cath lab and they're not really sure how you do a transfer. – Emergency Physician, Facility A*

*We have a little typed out sheet on STEMI protocol. I usually orient that person to that sheet, which has like a little algorithm on what to do for the patient. Then, we talk about it, verbally talk about it, because their opportunity to see a STEMI during orientation may not happen. The main thing is, I give them that algorithm and say, "This is what we need to do and what needs to happen." Then, a lot of times, if we have a STEMI, pretty much every employee is involved. A new person can learn that way. If somebody you know that has not ever taken care of one, you say, "Hey, we got a STEMI, come on in here so you can see what's going on and what we do." You get that person in there so they can get some hands on. – Nurse Supervisor, Facility C*

Our next Resource subtheme was Experience and Team Composition. There was a clear emphasis on the compatibility of individuals within the larger organization, for the ED as a whole, and particularly as a member of the team caring for the patient with STEMI. Team composition also includes ensuring adequate experience and training of the team.

*When you start staffing your EDs with people who aren't [emergency medicine] trained, you see a huge amount of variation in their management of STEMI patients and what happens – Emergency Physician, Facility A*

*They need to, for one, become comfortable with the STEMI process, the protocols, and not panic… I think the more transfers you're involved in, the smoother it goes. When you're not involved in that many, I think the paperwork's a little overwhelming. – Nurse, Facility A*

*The people who have the experience, it helps. Ideally, your quarterback is your experienced, knowledgeable physician, who's familiar with the systems and protocols in place and can navigate those appropriately, and who has every cardiologist's cell phone on their cell phone. – Emergency Physician, Facility C*

The last subtheme was Characteristics of Resources. Interviewees emphasized the availability and reliability of physical resources like rooms, computers, fax, and EKG machines.

*The main breakdowns that I have seen are when [EMS] has too many units out that are unavailable. That made it like … Because we can't send the STEMI in a convalescent ambulance service, but that would be the main thing that would delay it. – Emergency Physician, Facility A*

*The problems with STEMIs are as we never know, the availability of the cath lab. – Nurse, Facility B*

## Discussion

As the number and frequency of inter-facility transfers for STEMI increase, staff and provider perceptions of the factors that affect timeliness provide an enriched understanding of the delays and variability observed in quantitative research. To our knowledge, this is the first study to take an inter-organizational examination of staff and provider perspectives actively involved in the inter-facility transfer of STEMI patients. The present study identifies three key findings for improving STEMI regionalization and interfacility transfers. First, protocols are a useful and largely institutionalized component of STEMI transfers, but as our findings show, can break down in systematic ways. Second, substantial barriers to inter-organizational communication exist both during and after transfers and represent major opportunities for coordination and process improvement. Finally, the use, availability, composition, and experience of both human and physical resources were perceived as important characteristics in inter-facility STEMI transfers.

A qualitative approach enhances our understanding of how to achieve a system of effective and timely transfers. Pervasive throughout our interviews was an overarching recognition of the importance of widespread awareness of and responsiveness to protocols for shaping effective, timely STEMI transfer. However, notable deficiencies in the use of protocols exist. These include the practical use of protocols during transfers and that staff do not feel the need to “pull out a piece of paper” and follow each of the steps. Ambiguity in dealing with specific situations combined with process variability depending upon which nurse and physician are working, results in diminished standardization of the transfer process. Future protocol development and refinement should consider how protocols are used in practice, what the ideal use of protocols is (e.g., reference vs. use during transfers). Comparable to the use of checklists, development of protocols may need to be focused on key activities necessary to enhance usability rather than being exhaustive [16, 17].

Performance benchmarks were also identified as particularly effective at raising internal awareness of performance goals as well as encouraging and motivating staff. However, the transferring ED lacked familiarity with each other’s performance metrics (i.e., how each organization evaluates their performance). Collaboration, potentially in the form of enhanced communication across organizations regarding performance is a potential opportunity to align goals and enhance coordination of these distinct organizations.

Communication was central to safe and effective transfers. We identified three categories of communication surrounding and during transfers that interviewees saw as compromising their ability to transfer and initiate care in an effective and timely manner - uninitiated, post-event, and the quality of the communication that does occur. Before and during transfers, uninitiated communication and the quality of communication appear to be most relevant. Uninitiated communication represents a potential opportunity to address anticipated or witnessed problems. Alternatively, low quality communication involved dropped, misdirected, and unnecessary communication that was reported to impede transfers and potentially compromise safety. Redundant communication, for example a nurse giving report twice, was a recurring and particularly frustrating source of inefficiency that delayed timely intervention during the transfer process.

Following transfers, lack of post-event communication primarily involved the feedback of patient outcomes to the emergency care teams. Staff identified that communication after transfers, such as patient outcome reports, was an important source of communication viewed as particularly valuable to the transferring ED staff. Patient outcome reports and feedback on transfer performance were reported as rarely given, highly desired, and an opportunity for learning, professional satisfaction, and process improvement. Notably, reports of patient outcomes are viewed by ED providers as important to professional satisfaction, process improvement, clinical education, and medical error prevention [3, 4]. This latter finding builds on the broader picture of collaboration and coordination across organizations identified in the discussion of performance benchmarks described above. These need not solely occur during the transfer but can occur after as well. A Cochrane review on Audit and Feedback found that it is an effective mechanism for provider change [18] and is low-intensity approach to develop a learning health system. Moreover, considering the high degree of burnout among the emergency care workers [19–21], the provision of patient outcomes feedback may be protective [22].

Finally, interviewees recognized the role that both human and facility resources play in safe and effective transfers. Team composition and introduction to the team combined with the availability and capacity of physical resources, are relevant dimensions of ED performance during ED transfers. Interestingly, the comments were not about the presence or lack of such resources, but rather the compatibility and reliability of these resources within the existing environment.

From these interviews, we have identified several actionable opportunities for improvement in STEMI transfers that are summarized in Table 2. Under Process and Protocols, we recommend that new or updated protocols should recognize that transfer protocols may not be used during the actual event. Highly detailed protocols may be useful references but will likely have limited use during the transfer. To ensure protocols are most helpful and used during an event, they should identify and emphasize the highest yield activities that are required for transfers. Next, while the promotion of performance benchmarks internally appears to raise awareness of expectations and are used as positive reinforcement, this also represents an opportunity for inter-organizational coordination by identifying mutually agreeable benchmarks that can be shared and communicated to identify successes and opportunities for improvement. Identifying and aligning inter-organizational goals for transfer through shared discussion and collaboration may foster performance improvement that is better aligned with patient-centered goals.

**Table 2:** Summarized recommendations to enhance the effectiveness of inter-facility transfers for STEMI identified from interviews

Theme	Recommendation
Process and Protocols	1. Develop and review STEMI transfer protocols to identify a limited number of highly important actions 2. Identify and agree upon a limited set of performance goals for inter-organizational performance that are regularly shared
Communication	3. Develop formal mechanisms for patient outcome reporting for STEMI patients transferred that include the entire treating team at all locations 4. Shared electronic medical records between organizations
Resources	5. Recruit individuals focusing on experience and organizational fit as desirable characteristics 6. Develop realistic formal training for inter-facility transfers (e.g., simulations) that emphasize teamwork and enhances shared familiarity with the processes

Inter-organizational coordination may be further reinforced by developing formal mechanisms for post-event communication or feedback internally and across organizations. Feedback should be timely to enhance event recall, include multiple staff roles involved at the time of the event, and could provide a platform for the staff member to detail successes or challenges during the event.

Last, our work did not identify the need for additional resources, but rather to focus on enhanced integration of existing resources through onboarding and team development. Our respondents noted that this was more likely to occur with the right composition of the team - experienced and flexible staff. Identifying potential characteristics in such staff during recruitment and emphasizing these traits during a formal onboarding and training program can help to ensure their use during actual transfers not only for STEMI but other time-sensitive conditions too.

### Limitations

Our findings should be considered in light of potential limitations. First, a qualitative approach conducted at three unaffiliated medical centers may limit generalizability because we were specifically trying to capture the detail and nuance of transfer processes in one region that is among the highest in cardiovascular disease in the U.S. [23] However, the number of interviewees (*N* = 43) and the breadth of roles interviewed in this study also provides a large, representative sample of staff working on these types of transfers. Moreover, a qualitative approach also captures more richness about specific processes as they unfold and enhances our understanding of facilitators and barriers to effective transfers. While initial subjects were identified through ED management which may bias our findings to the extent that management may be inclined to recommend individuals that would provide positive assessments, our data still contained both barriers and facilitators and examples of poor transfers. We further mitigated this potential bias by using snowball sampling to broaden the survey respondents beyond those recommended by ED management. Future research can ensure the robustness of our findings by using alternative approaches (e.g., random sampling) and methodologies (e.g., focus groups). We further mitigated potential selection bias by achieving theoretical saturation in our responses. While we were not able to interview all potential personnel and staff at transferring and receiving facilities (e.g., administration) we sought to understand perceptions from the standpoint of the primary staff and clinicians who make the decision to transfer such patients. Future research should build on our work to include a broader array of personnel and explore the downstream consequences of transfers. Next, our findings reflect the perceived influence of each theme on timeliness and subsequent patient outcomes. Future research will need to directly link these themes with patient and family experiences as well as their relationship with observed clinical and operational outcomes. Finally, interviews were subject to recall bias potentially limiting the accuracy of recalled events. Nevertheless, this work provides several key insights to further guide examination, development, and improvement of local and regional STEMI systems of care.

## Conclusions

In conclusion, our qualitative interviews identified a broad range of staff and clinical provider-perceived strengths and opportunities for enhancing the timeliness, safety, and quality of acute care at EDs that transfer STEMI patients.

## Supplementary information

**Additional file 1:.** Supplemental Figure 1. Semi-structured staff and clinical provider interview guide.

## Data Availability

The datasets generated and/or analyzed during the current study are not publicly available due to potential loss of confidentiality but are available from the corresponding author on reasonable request.

## Abbreviations

AHA: American Heart Association
COREQ: COnsolidated criteria for REporting Qualitative research
ED: Emergency Department
EKG: Electrocardiogram
ER: Emergency Room
IRB: Institutional Review Board
PCI: Percutaneous Coronary Intervention
STEMI: ST-Elevation Myocardial Infarction
VUMC: Vanderbilt University Medical Center
